# Evaluation of Granulocyte Colony-Stimulating Factor Effects on Treatment-Resistant Thin Endometrium in Women Undergoing *In Vitro* Fertilization

**DOI:** 10.1155/2014/913235

**Published:** 2014-02-12

**Authors:** Michał Kunicki, Krzysztof Łukaszuk, Izabela Woclawek-Potocka, Joanna Liss, Patrycja Kulwikowska, Joanna Szczyptańska

**Affiliations:** ^1^INVICTA Fertility and Reproductive Center, 00-019 Warszawa, Poland; ^2^Department of Obstetrics and Gynecological Nursing, Faculty of Health Sciences, Medical University of Gdansk, 80-952 Gdańsk, Poland; ^3^Department of Obstetrics and Gynecology, Faculty of Medical Sciences, University of Varmia and Masuria, 10-561 Olsztyn, Poland; ^4^INVICTA Fertility and Reproductive Center, 80-850 Gdańsk, Poland; ^5^Department of Reproductive Immunology and Pathology, Institute of Animal Reproduction and Food Research, Polish Academy of Sciences, 10-747 Olsztyn, Poland

## Abstract

The aim of the study was to assess the granulocyte colony-stimulating factor (G-CSF) effects on unresponsive thin (<7 mm) endometrium in women undergoing *in vitro* fertilization (IVF). We included thirty-seven subjects who had thin unresponsive endometrium on the day of triggering ovulation. These patients also failed to achieve an adequate endometrial thickness in at least one of their previous IVF cycles. In all the subjects at the time of infusion of G-CSF, endometrial thickness was 6,74 ± 1,75 mm, and, after infusion, it increased significantly to 8,42 ± 1,73 mm. When we divided the group into two subgroups according to whether the examined women conceived, we showed that the endometrium expanded significantly from 6,86 ± 1,65 to 8,80 ± 1,14 mm in the first group (who conceived) and from 6,71 ± 1,80 to 8,33 ± 1,85 mm in the second, respectively. There were no significant differences between the two subgroups in respect to the endometrial thickness both before and after G-CSF infusion. The clinical pregnancy rate was 18,9%. We concluded that the infusion of G-CSF leads to the improvement of endometrium thickness after 72 hours.

## 1. Introduction

Many factors could have impact on the *in vitro* fertilization-embryo transfer IVF-ET success. The main independent variables are: the age of women, antimullerian hormone (AMH) concentrations, number of embryos transferred and their quality [1]. It was also demonstrated that endometrial thickness <7 mm negatively affected pregnancy rate [2, 3]. Moreover, Sharkey showed that immunological mechanisms in the endometrium are very important and crucial in the implantation process [4]. Some investigators demonstrated that the growth factors, hormones, and cytokines, which are produced by decidual cells, are involved in the implantation process [5]. Preliminary studies demonstrated that G-CSF stimulated neutrophilic granulocyte proliferation and differentiation, acted on macrophages of decidual cells, and finally affected the implantation [6, 7]. What is more, known and reported immune effects of G-CSF are recruitment of dendritic cells, promoting Th-2 cytokine secretion, activating T regulatory cells, and also stimulation of various proangiogenic effects [7, 8]. On the other hand, the receptor for GCSF is expressed by the trophoblastic cells and by human luteinized granulosa cells [9, 10]. It was also stated that G-CSF prevented repeated miscarriages and implantation failures [11, 12]. In the last two years, Gleicher et al. presented two clinical studies with limited number of participants regarding the usefulness of G-CSF treatment in endometrium expansion in women who had previously cancelled cycles because of the unresponsive endometrium [13, 14]. Taking into account all these data, the aim of the study was to examine G-CSF effects on unresponsive thin (<7 mm) endometrium in women undergoing IVF.

## 2. Materials and Methods

We presented a series of 37 patients who have undergone IVF procedure.

The inclusion criteria were as follows:

- women aged 18–45 years,
- previously cancelled at least one cycle because of thin unresponsive endometrium (<7 mm) during IVF programs,
- inadequate thin endometrium (<7 mm) on the day of hCG injection,
- the lack of contraindications for G-CSF treatment (sickle cell disease, chronic neutropenia, known past or present malignancy, renal insufficiency, upper respiratory infection, pneumonia, and congenital fructose intolerance),
- personal agreement for such still experimental therapy,
- no prenatal genetic screening,
- no Asherman's syndrome, fibroids, and polyps in diagnostic hysteroscopy.

The study protocol was approved by the Institutional Review Board of Varmia and Masuria, Olsztyn, Poland, and written informed consent was given by each participating women.

The primary end point was the endometrial thickness measured in transvaginal sonography. The second point was clinical pregnancy after embryo transfer. Identification of an intrauterine gestational sac by transvaginal ultrasonography together with an increasing serum *β*-hCG constituted a clinical state of pregnancy. All included women had previously long agonist protocol in all cases.

During the study, all patients received oral contraceptive pill (OCP) starting on days 2–5 of spontaneous menses of the cycle prior to the treatment cycle. The OCP contained 0.03 mg ethinyl estradiol (E2) and 0,15 mg desogestrel Ovulastan (Polfa, Poland). OCPs were taken daily for 21 days. Patients were administered s.c. GnRH agonist 0.1 mg gonapeptyl (Ferring, The Netherlands) daily. The agonist was started 4-5 days before discontinuation of the OCP. When desensitization was achieved as evidenced by plasma E2 levels of <50 pg/mL [15], daily s.c. injection of highly purified menotropin (Menopur, Ferring, The Netherlands) was commenced. When at least two follicles reached a minimum of 17 mm diameter, 5000 U of hCG (Choragon Ferring, The Netherlands) was applied. Transvaginal pick-up was performed 36 h hours after hCG administration under transvaginal sonography.

The infusion of G-CSF was made according to Gleicher et al.'s procedure with full bladder before transfer [14]. Frydman catheter was introduced to the uterine cavity. We infused under ultrasound guidance 30 mL (300 mg/1 mL) of G-CSF (Neupogen, Filgastrim, Amgen Inc., Thousand Oaks, CA, USA).

Endometrium was reassessed after 72 hours. If it expanded, all transfers were performed by two doctors. When endometrium was below 7 mm after G-CSF, women could choose two options: to have blastocyst transfer, despite inadequate endometrium, or to cancel the cycle. In that case, embryos were frozen. The number of embryos transferred varied from one to three.

## 3. Statistical Analysis

Continuous variables were presented as mean ± SD; categorical variables were presented by ratio. Differences between dependent variables (before and after) were checked by paired *t*-test. Differences between independent variables were checked by *t*-test. *P* < 0.05 was considered statistically significant. The statistical package STATISTICA (data analysis software system), version 10.0 (StatSoft Inc., Tulsa, OK; http://www.statsoft.com/), was used for data analysis.

## 4. Results

The baseline characteristics of participants are shown in Table 1.  Table 2 presents endometrial thickness in women before and after infusion of G-CSF who had IVF-ET. In all the subjects at the time of infusion of G-CSF, endometrial thickness was 6,74 ± 1,75 mm, and, after infusion, it increased to 8, 42 ± 1,73 mm (*P* < 0.001). When we divided the group into two subgroups according to whether they conceived, we showed that the endometrium increased from 6,86 ± 1,65 to 8,80 ± 1,14 mm in the first one and from 6,71 ± 1,80 to 8,33 ± 1,85 mm in the second one (*P* < 0.001). There were no significant differences between the two subgroups in respect to the endometrial thickness both before (*P* = 0.84) and after infusion (*P* = 0.86). The clinical pregnancy rate was 18.9%. Seven women conceived and delivered. Four women had one single sac and one single term birth. We recorded three women with two gestational sacs. One of them had preterm single live birth, whereas the second delivered prematurely single birth—the baby had intrauterine death—and the third had term birth of twins. No triplets were recorded.

All the women were supplied with low-dose aspirin, sildenafil citrate (Viagra), or both in the treatment cycle and in previous cycles.

Three out of seven women who conceived after GCSF were pregnant once before entering the study; one woman was pregnant twice. Eight out of thirty in subgroups who did not conceive were pregnant once, whereas four women twice and one women three times, respectively.

The mean endometrial thickness in previous failures was 5,75 mm ± 1,0 mm for all women; when we divided women according to whether they conceived, the mean endometrial thickness was 6,45 ± 0,38 mm for those who conceived and 5,95 ± 0,76 mm for those who did not conceive, respectively.

## 5. Discussion

It has been demonstrated before that <1% of women have thin endometrium [2, 16]. The thin unresponsive endometrium is still the unresolved clinical problem. There are inconclusive data regarding the diameter of so-called thin endometrium. Some investigators stated that that the pregnancy occurs when endometrium reaches more than 7 mm and others that more than 9 mm [2, 3, 15, 16]. However, there are also data in the literature that endometrium 5–8 mm is inadequate [17]. Several methods were proposed, to increase thin endometrium in women undergoing IVF. These therapies included tocopherol, pentoxifylline, low-dose aspirin, sildenafil citrate, and estradiol administration [16, 18, 19].

The embryos of patients with thin endometrium have to be frozen, which leads to the real clinical dilemma for doctors. On the other side, in some investigations, there was no correlation between the IVF outcome and endometrium thickness [20, 21]. In the pilot study of Gleicher et al., the authors showed preliminary clinical report regarding the role of G-CSF on endometrium expansion in women with unresponsive endometrium [13]. In this case report, the data of four patients infused with G-CSF into the uterus were demonstrated. All these patients finally conceived. Two years later, the same group of authors described 21 patients with inadequate thin endometrium infused with G-CSF. As a result, 19.1% ongoing clinical pregnancy rate was observed. The findings of Gleicher et al. provided evidence that G-CSF could be promising agent in the treatment of women with thin unresponsive endometrium [14].

In our study, we analyzed 37 women who underwent IVF-ET after G-CSF infusion. The clinical pregnancy rate was very similar to Gleicher study [14]—18.9%. We found that the endometrium significantly increased after infusion of G-CSF when we analyzed all the examined women and when we divided them according to the conception success. The increase of endometrium thickness was greater in group of women who conceived but the difference between groups was not statistically significant. These observations were in accordance with the study presented by Gleicher et al. [14]. In that pilot data, the endometrium increased also in all women but more in the subgroup of women who conceived. In contrast to Gleicher et al.'s study, our population was younger (34,6 versus 40.5 years) and had higher AMH concentrations (4,2 versus 1,5 ng/mL). Another difference was the time interval between G-CSF infusion and the first reassessment of the endometrium; in Gleicher et al. [14] study, it was 48 hours in contrast to 72 hours in our study. However, the question what is the appropriate interval between infusion and second reassessment of endometrium is still open. Secondly, we are still not sure how many times G-CSF should be applied. In Gleicher et al.'s study [14], three patients (14.3%) reached the minimal thickness after the second infusion of G-CSF. In contrast, in our study, we infused G-CSF only once. We also do not know the extent to which the increase of endometrial thickness is the result of G-CSF function or the synergistic effect of added low-dose aspirin and to the protocol. This supplementation was routinely applied in our study. There are opposing results with aspirin in unselected IVF patients on the endometrium thickness and pregnancy rates [22, 23].

Our study is not without limitations. Firstly, we did not have a control group which received placebo. Thus, the changes of endometrial thickness could be observed only before and after infusion and between subgroups of women who conceived or not. Secondly, the subgroup of women who conceived was very small. Thirdly, we applied aspirin and/or sildenafil citrate which also could have a positive effect on endometrial thickness. For example, aspirin attenuates placental apoptosis, and this could be a possible explanation of how aspirin is beneficial, even in the absence of endometrial or oocyte improvement [24]. We can only speculate that the other factors could have impact on endometrial thickness. For example, we did not measure antiphospholipid antibodies [25]. The presence of them could have influenced endometrial thickness during low-aspirin treatment. But we do not believe that this could have essential impact on our results. We think that taking into account all previous failures the G-CSF effect could play the main role in our study. One should note that women who conceived were younger than women who did not but the difference between groups was not statistically significant.

Therefore, the final assessment on how G-CSF affects the expansion of endometrium thickness remains open until prospectively controlled studies would be performed.

To date, no final conclusions have been also drawn regarding which delivery system is better. Despite the aim of our study, we did not compare the assessment of adverse events and did not record any adverse effect during G-CSF infusion. However, it was demonstrated before that the treatment with G-CSF could lead to bone pain, general fatigue, headaches, insomnia, anorexia, nausea, and/or vomiting [26]. Additionally dyspnea, chest pain, hypoxemia, diaphoresis, anaphylaxis, syncope, and flushing were recorded [27]. There is also a question on how to properly counsel the patients who did not conceive and still have thin unresponsive endometrium despite G-CSF infusion. However, we showed some possibilities to the patients.

In summary, we showed that, in women who had thin endometrium in the previous IVF cycles, the infusion of G-CSF increases the endometrial thickness. Additionally, the expanding of endometrial thickness was observed after 72 hours. Because of the limited number of women and no control group, our conclusions are limited. We should also remember that the threshold is different in many other studies; thus, clinical pregnancy was observed even in women with endometrium <4 mm [28]. We think that, despite the obvious limitations, our data are important for doctors and couples seeking fertility assistance. However, further studies are needed in this field.

## Figures and Tables

**Table 1:** Baseline patient characteristics and IVF cycle characteristics in women with thin endometrium.

Characteristic	All women *n* = 37	Women who conceived *n* = 7	Women who did not conceive *n* = 30
Age (years)	34.68 ± 4.13 (35)	32.14 ± 2.79 (33)	35.32 ± 4.20 (36)
Primary infertility diagnosis	24/37 (64.86%)	4/7 (57.14%)	20/30 (66.67%)
Secondary infertility diagnosis	13/37 (35.14%)	3/7 (42.86%)	10/30 (30.33%)
BMI (kg/m^2^)	23.09 ± 2.78 (23.31)	21.89 ± 1.37 (22.15)	23.36 ± 2.97 (23.85)
FSH (mIU/mL)	7.18 ± 1.91 (7.3)	6.87 ± 1.40 (7)	7.31 ± 2.19 (7.60)
AMH (ng/mL)	4.28 ± 3.29 (3.8) 0.1–12.8	6.37 ± 4.17 (4.60) 1–12.8	3.78 ± 2.91 (3.4) 0.1–10.8
Cycles	3.46 ± 2.23 (3.00) 1–11	3.29 ± 1.80 (3.00) 2–7	3.5 ± 2.35 (3.00) 1–11

Continuous variables are shown as mean ± standard deviation.

Categorical variables are shown as ratio.

**Table 2:** Endometrial thickness in women before and after infusion of G-CSF who had IVF-ET.

Characteristic	All women *n* = 37	Women who conceived *n* = 7	Women who did not conceive *n* = 30
Endometrial thickness before G-CSF-infusion	6.74 ± 1.75^1^	6.86 ± 1.65^2^	6.71 ± 1.80^3^
Endometrial thickness after G-CSF infusion	8.42 ± 1.73^1^	8.80 ± 1.14^2^	8.33 ± 1.85^3^
Endometrial thickness (Δ)	1.68 ± 1.05	1.94 ± 0.99^4^	1.62 ± 1.07^4^

Data are shown as mean ± standard deviation.

*P* value for two dependent samples (before versus after).

^1^ *P* < 0.001.

^2^ *P* = 0.0020.

^3^ *P* < 0.0001.

*P* value for two independent samples (women who conceived versus women who did not conceive).

^4^ *P* = 0.8481.

^5^ *P* = 0.2444.

^6^ *P* = 0.4650.
